# Anti-Hyperuricemic Effect of 2-Hydroxy-4-methoxy-benzophenone-5-sulfonic Acid in Hyperuricemic Mice through XOD

**DOI:** 10.3390/molecules23102671

**Published:** 2018-10-17

**Authors:** Tianqiao Yong, Dan Li, Muxia Li, Danling Liang, Xue Diao, Chenling Deng, Shaodan Chen, Yizhen Xie, Diling Chen, Dan Zuo

**Affiliations:** 1 State Key Laboratory of Applied Microbiology Southern China, Guangdong Provincial Key Laboratory of Microbial Culture Collection and Application and Guangdong Open Laboratory of Applied Microbiology, Guangdong Institute of Microbiology, Guangzhou 510070, China; 13751717863@163.com (D.Li); 13760722769@163.com (M.L.); liangdanling@hotmail.com (D.Liang); xuediao92@126.com (X.D.); 15820115385@163.com (C.D.); chenshaodan@126.com (S.C.); diling1983@163.com (D.C.); 2 R & D Department, Guangdong Yuewei Edible Fungi Technology Co., Guangzhou 510663, China; 3 College of Chinese Materia Medica, Guangzhou University of Traditional Chinese Medicine, Guangzhou 510006, China; 4 Guangzhou Institutes of Biomedicine and Health, Chinese Academy of Sciences, Guangzhou 510530, China; zuo_dan@gibh.ac.cn

**Keywords:** 2-hydroxy-4-methoxybenzophenone-5-sulfonic acid, hyperuricemia, xanthine oxidase, organic anion transporter 1, toxicity

## Abstract

Conventionally, benzophenone-type molecules are beneficial for alleviating the UV exposure of humans. More importantly, various compounds with this skeleton have demonstrated various biological activities. In this paper, we report the anti-hyperuricemic effect of the benzophenone compound 2-hydroxy-4-methoxybenzophenone-5-sulfonic acid (HMS). Preliminarily, its molecular docking score and xanthine oxidase (XOD) inhibition suggested a good anti-hyperuricemic effect. Then, its anti-hyperuricemic effect, primary mechanisms and general toxicity were examined on a hyperuricemic mouse model which was established using potassium oxonate and hypoxanthine together. HMS demonstrated a remarkable anti- hyperuricemic effect which was near to that of the control drugs, showing promising perspective. General toxicity was assessed and it showed no negative effects on body weight growth and kidney function. Moreover, anti-inflammatory action was observed for HMS via spleen and thymus changes. Its anti-hyperuricemic mechanisms may be ascribed to its inhibition of XOD and its up-regulation of organic anion transporter 1 (OAT1) and down-regulation of glucose transporter 9 (GLUT9).

## 1. Introduction

Due to their significant absorption of ultraviolet radiation (UV), UV filters extensively dominate sunscreen formulations in many products, such as cosmetics, plastics, packages and films [1] and are even utilized as DNA photosensitizers and photocatalysts [2]. Of all these, the most important ones are benzophenone-type chemicals, since ultraviolet (UV) irradiation can excite them to undergo an ultrafast conversion from singlet to triplet states, which is the very property that is exploited in some of the above applications. Due to their unique and typical photochemical properties, they also serve as archetypal systems for investigating the fundamental photochemistry of aromatic ketones [3].

Besides the main benefit of benzophenone-type chemicals of alleviating human UV exposure, they are also considered as endocrine disruptors [1,4]. Moreover, many references have reported their biological risks since they readily permeate into the body [5] and are easily released into the ecosystem, exerting neurotoxicity [6,7], genotoxicity [8], immunosuppression [9] and estrogenic activity [10]. Moreover, at high concentrations benzophenone-type chemicals are known allergens and potential carcinogens. However, they were evaluated as low-risk chemicals in the aquatic ecosystem or for humans [11] due to their negligible concentrations [12].

**Figure 1:**
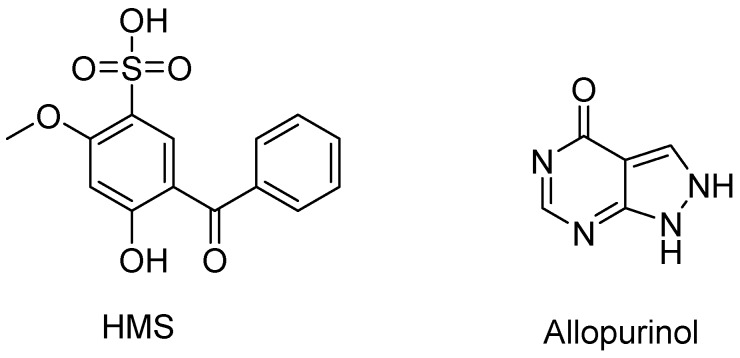
Structures of 2-hydroxy-4-methoxybenzophenone-5-sulfonic acid (HMS) and allopurinol.

In addition to the above, benzophenone-type compounds [13,14] from natural products and synthesized ones have demonstrated important and valuable biological activities, with anti-inflammatory, antimicrobial, cytotoxic activities [15,16] and anti-angiogenesis effects [17] as examples. Nemorosone has anti-proliferative activity by targeting Akt/PKB against LAN-1 parental cell line [18] and cytotoxic activity against HeLa, eHep-2, PC-3 and U251 cancer cells [19,20]. Besides, a series of synthesized benzophenones exhibited anti-proliferative activity against DLA cells [21]. In our studies, some of them exhibited good XOD inhibitory activities, such as those substituted by hydroxyls [22] and thiazolidinone groups [23], making them promising as potential anti-hyperuricemic agents, although their in vivo anti-hyperuricemic effects were not reported. Of benzophenone-type chemicals, 2-hydroxy-4-methoxybenzophenone-5-sulfonic acid (HMS, Figure 1) is the most popular UV-filter, with a maximum permitted concentration in cosmetics currently set at 6% (USA), 5% (EU and China), and 10% (Japan) [1], which was much higher than doses in drugs (μM in vitro or mg/kg in vivo). Its abundance and inexpensive characteristics render it promising. However, its anti-hyperuricemic activity has not been mentioned.

In this paper, HMS, a representative of the benzophenone-type derivatives, was screened as a top candidate against xanthine oxidase (XOD), showing promising perspectives and we investigated its anti-hyperuricemic effect first in vitro and then in vivo. Firstly, we analyzed its interaction against XOD in detail by molecular docking using as positive control oxypurinol. Then, its in vitro inhibitory activity was assayed against XOD. To evaluate if it was effective in vivo, a hyperuricemic mouse model was established, where serum uric acid (SUA) was recorded to examine its anti-hyperuricemic efficacy. Urine uric acid (UUA), XOD activity in serum and liver and key transporters in kidney and gastrointestinal tract, such as organic anion transporter 1 (OAT1), glucose transporter 9 (GLUT9) and concentrative nucleoside transporter 2 (CNT2), were examined to explore its possible mechanisms. Also, urea nitrogen and creatinine in blood and urine, body weight growth and inner organ coefficients were measured to evaluate its general toxicity.

## 2. Results

### 2.1. Hypouricemic of HMS In Vitro against XOD by Molecular Modeling and Enzymatic Activity Assays

**Figure 2:**
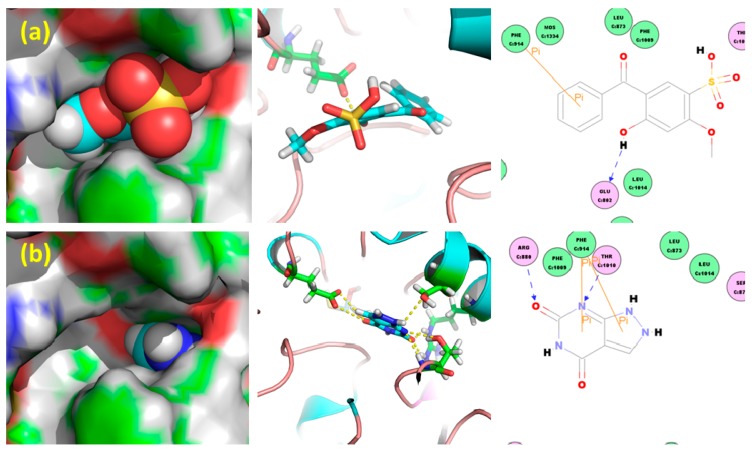
Docked structures of (**a**) HMS and (**b**) oxypurinol to XOD (PDB: 1FIQ).

In order to assess HMS’s potential inhibition against XOD (PDB: 1FIQ), molecular docking was performed and shown in Figure 2. In comparison to the positive control oxypurinol which is almost buried in the inner active tunnel of XOD, HMS’s two cyclic moieties were buried into the inner tunnel and its two substituents attached to one aromatic moiety sited and occupied the outer tunnel.

Thus, HMS served as a nail, anchoring into the active tunnel of XOD, while oxypurinol acts as a block. Oxypurinol showed a -Cdocker_Energy of 2.60 kcal/mol and a -Cdocker_Interaction_Energy of 28.84 kcal/mol, while HMS depicted a -Cdocker_Energy of 11.28 kcal/mol and a -Cdocker_Interaction_Energy of 32.73 kcal/mol, which were some higher than these items of oxypurinol, implying the possibility of better inhibition against XOD.

**Figure 3:**
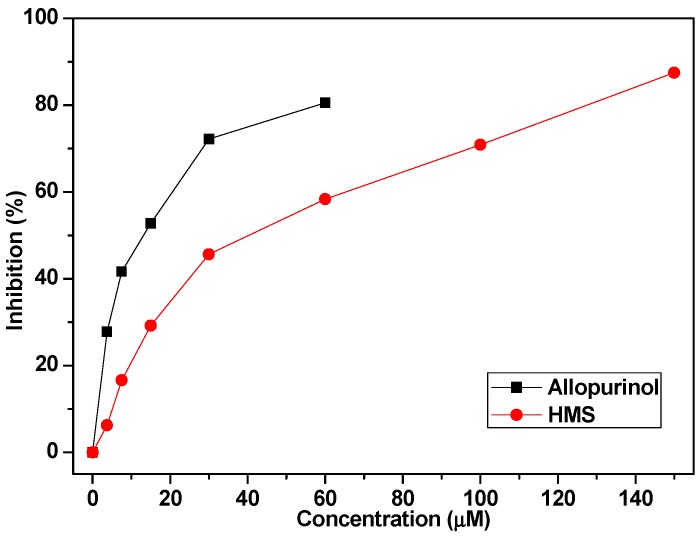
XOD inhibition of HMS.

The in vitro inhibitory activity of HMS was assayed using allopurinol as positive control (Figure 3), wherein, allopurinol demonstrated an IC_50_ of 11.40 μM; while HMS 36.11 μM, which was some higher than that of allopurinol. This was not consistent with the predicted result.

### 2.2. Hypouricemic Effect of HMS In Vivo on a Hyperuricemic Mouse Model

**Figure 4:**
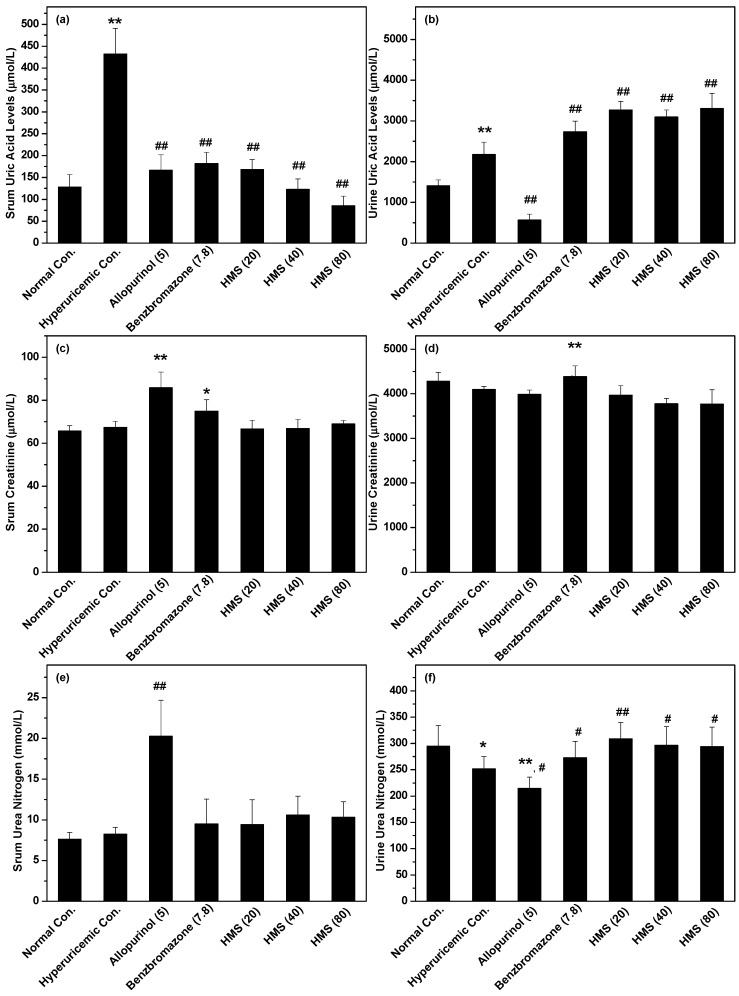
Effects of HMS on the key physiologic parameters in hyperuricemic mice: (**a**) SUA, (**b**) UUA, (**c**) serum creatinine, (**d**) urine creatinine, (**e**) serum BUN, (**f**) urine BUN. Data were expressed as mean ± SD; n = 8. Statistical analysis by one-way ANOVA followed by two-tailed Student’s *t*-test; * *p* < 0.05, ** *p* < 0.01 *versus* the normal control; ^#^ *p* < 0.05, ^##^ *p* < 0.01 *versus* hyperuricemic control.

The hypouricemic effect of HMS was examined in vivo on hyperuricemic mice and SUA was recorded (Figure 4a) to evaluate its efficacy. As depicted, models were built successfully, where SUA of hyperuricemic control was enhanced to 432.31 ± 58.7 μmol/L (*p* < 0.01) from normal control (128.28 ± 28.57 μmol/L).

Positive allopurinol and benzbromarone controls depressed it to 166.61 ± 35.41 and 181.85 ± 25.48 μmol/L (*p* < 0.01), respectively, which further suggested the success of the hyperuricemic models. Especially for HMS, it suppressed SUA of hyperuricemic mice to 168.38 ± 22.44, 122.95 ± 23.81 and 85.57 ± 21.71μmol/L (*p* < 0.01) at 20, 40 and 80 mg/kg which were near or even lower than normal control, showing crystal anti-hypouricemic effects.

Since UUA impacts SUA directly through kidney, UUA was assessed to evaluate if HMS lowered SUA through elevating UUA (Figure 4b). As anticipated, hyperuricemic control demonstrated an enhanced UUA (2177 ± 301 μmol/L) in comparison to normal control (1408 ± 142 μmol/L, *p* < 0.01). On the other hand, allopurinol, a clinic XOD inhibitor, decreased UUA (568 ± 146 μmol/L, *p* < 0.01) in comparison to hyperuricemic control. The traditional uriuric, benzbromarone, increased UUA to 2735 ± 257 μmol/L (*p* < 0.01). Similarly, HMS at three doses enhanced UUA to 3269 ± 208, 3099 ± 169 and 3304 ± 370 μmol/L (*p* < 0.01), showing some analogous effects to benzbromarone.

Serum creatinine is an important indicator for renal health. Hyperuricemic control demonstrated a slight higher creatinine (67.42 ± 2.73 μmol/L, *p* < 0.05) than normal control (65.81 ± 2.37 μmol/L, Figure 4c). Allopurinol and benzbromarone enhanced it to 85.85 ± 7.26 and 74.91 ± 5.35 further. HMS at low, middle and high doses (66.66 ± 3.99, 66.93 ± 4.06 and 69.01 ± 1.63 μmol/L) showed similar creatinines to normal control.

We also recorded the urine creatinine levels corresponding to the serum result. As expected, hyperuricemic control (4099 ± 67 μmol/L, *p* < 0.05) was slight lower than normal control (4283 ± 199 μmol/L, Figure 4d). Allopurinol further decreased urine creatinine to 3988 ± 95 (*p* < 0.05). Benzbromarone enhanced it to 4385 ± 241 μmol/L (*p* < 0.05). HMS reduced it to 3971 ± 214, 3777 ± 122 and 3770 ± 321 μmol/L.

Serum BUN is a frequently used index to evaluate renal function. In this animal experiment, hyperuricemic control depicted higher serum BUN (8.27 ± 0.83 mmol/L, *p* < 0.05) than normal control (7.63 ± 0.82 mmol/L), since some negative impacts of PO on renal (Figure 4e). Allopurinol impaired renal further, where it raised serum BUN of hyperuricemic mice to 20.27 ± 4.41 mmol/L (*p* < 0.01). Benzbromarone (9.51 ± 3.04 mmol/L, *p* > 0.05) did not show significant difference in comparison to hyperuricmic control. Similar effects were observed for HMS at three doses, which presented serum BUN at 9.42 ± 3.05, 10.59 ± 2.3 and 10.34 ± 1.87 mmol/L (*p* > 0.05).

As a corresponding indicator to serum BUN, urine BUN was recorded (Figure 4f). Wherein, urine BUN of hyperuricemic control (252 ± 23, *p* < 0.05) and allopurinol (215 ± 21, *p* < 0.01) were lower than normal control (295 ± 39). But benzbromarone (273 ± 31) and HMS at 20, 40 and 80 mg/kg (309 ± 31, 297 ± 35 and 294 ± 37, respectively) enhanced urine BUN from hyperuricemic control (*p* < 0.05 or 0.01) and this parameter of the four groups were observed to have no significance (*p* > 0.05) when they were compared to normal control.

**Figure 5:**
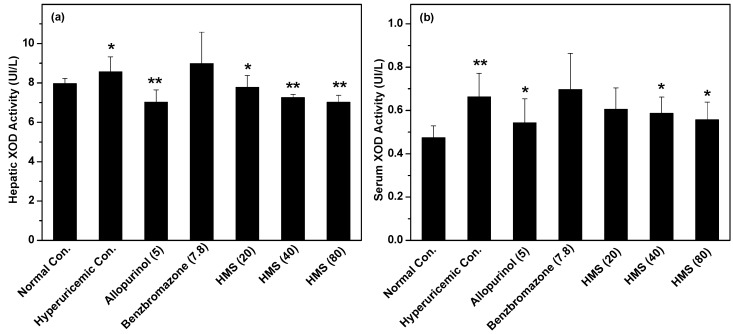
The influence of HMS against XOD in vivo: (**a**) hepar and (**b**) serum. Data were expressed as mean ± SD; n = 8. Statistical analysis by one-way ANOVA followed by two-tailed Student’s *t*-test; * *p* < 0.05, ** *p* < 0.01 versus the normal control.

The influence of HMS on XOD in liver was recorded in vivo (Figure 5a) using liver collected after the animal experiment. Since the intake of large amounts of HX, XOD activity of hyperuricemic control (8.56 ± 0.76 U/L, *p* < 0.05, Figure 4a) was elevated from normal control (7.97 ± 0.26 U/L). Due to allopurinol’s inhibitory effect, allopurinol reduced it to (7.02 ± 0.63 U/L, *p* < 0.01) from that of hyperuricemic control. This impact had not been observed for benzbromarone (8.99 ± 1.59 U/L). For HMS, XOD activities were suppressed to 7.77 ± 0.6, 7.25 ± 0.17 and 7.01 ± 0.36 U/L (*p* < 0.05 or 0.01), comparing to hyperuricemic control. This was consistent with the in vitro results.

On the other hand, HMS may interact with serum XOD firstly since it enters into the blood before reaching the liver, so serum XOD activities were recorded (Figure 5b). As expected, hyperuricemic control (0.66 ± 0.10 U/L, *p* < 0.01) exhibited higher serum XOD activity than normal control (0.47 ± 0.05 U/L). Allopurinol decreased serum XOD activity to 0.54 ± 0.11 U/L (*p* < 0.05) in hyperuricemic mice. Similarly, HMS at middle- and high- doses reduced it to 0.58 ± 0.07 and 0.55 ± 0.08 U/L (*p* < 0.05). This was consistent with the in vitro results.

**Figure 6:**
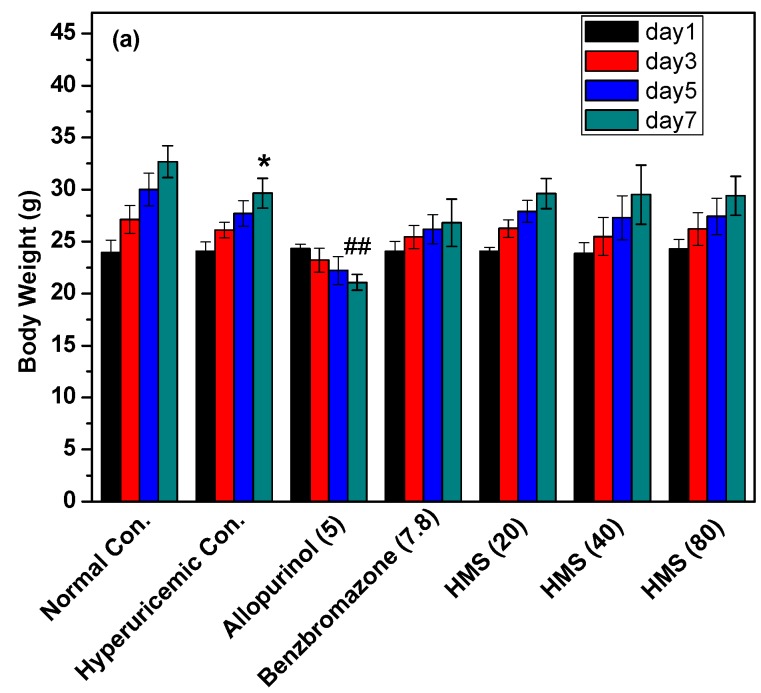

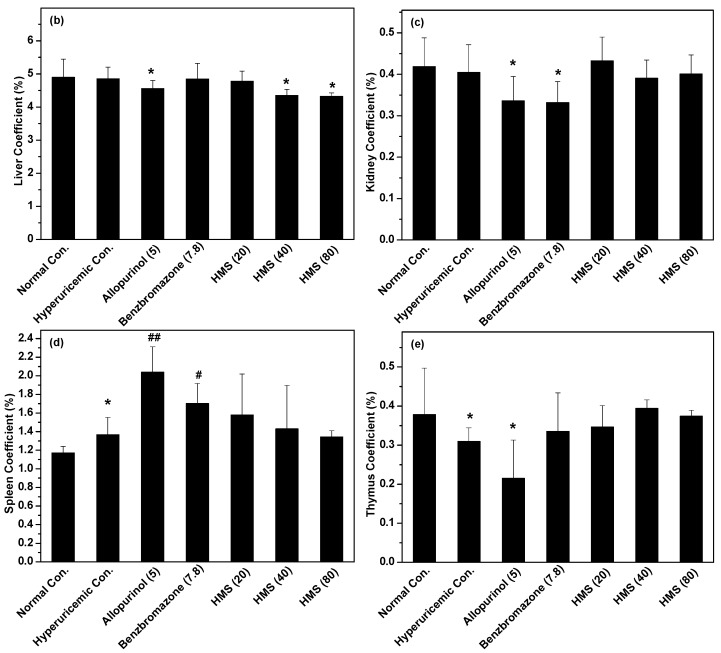
Impacts of allopurinol, benzbromarone and HMS on (**a**) body weight, (**b**) liver (**c**) kidney, (**d**) spleen and (**e**) thymus. Data were expressed as mean ± SD; n = 8. Statistical analysis by one-way ANOVA followed by two-tailed Student’s *t*-test; * *p* < 0.05 versus the normal control; ^#^ *p* < 0.05 or ^##^ *p* < 0.01 versus hyperuricemic control.

To evaluate the general toxicity of HMS, body weights and inner organ coefficients were recorded (Figure 6). At a glance, PO and HX used for model establishment suppressed body weight growth (*p* < 0.05) in hyperuricemic control in comparison to normal control (Figure 6a). Allopurinol reduced body weight growth further (*p* < 0.01) in comparison to hyperuricemic control. For HMS, there was no significant difference observed in comparison to hyperuricemic and normal controls.

On liver coefficient, there was no significant difference for hyperuricemic and benzbromarone controls and HMS at 20 mg/kg when they were compared with normal control (Figure 6b). However, allopurinol control and HMS at 40 and 80 mg/kg showed lower liver coefficients than normal control (*p* < 0.05), implying some negative impacts on liver function.

On kidney coefficient, PO and HX for model making had not impact kidney coefficient significantly (Figure 6c). However, allopurinol and benzbromarone reduced kidney coefficient (*p* < 0.05). This phenomenon had not been observed for HMS at three doses.

Spleen coefficient reflects the inflammatory state of the body. The spleen coefficient of hyperuricemic control was higher than that of normal control (*p* < 0.05, Figure 6d). Allopurinol and benzbromarone elevated spleen coefficients (*p* < 0.05 or 0.01), comparing to hyperuricemic control, but no significant difference was observed for HMS in comparison to hyperuricemic control.

PO and HX decreased thymus coefficient and allopurinol reduced it further (*p* < 0.05, Figure 6d). HMS restored the declined thymus coefficient of hyperuricemic mice to normal level (*p* < 0.05).

**Figure 7:**
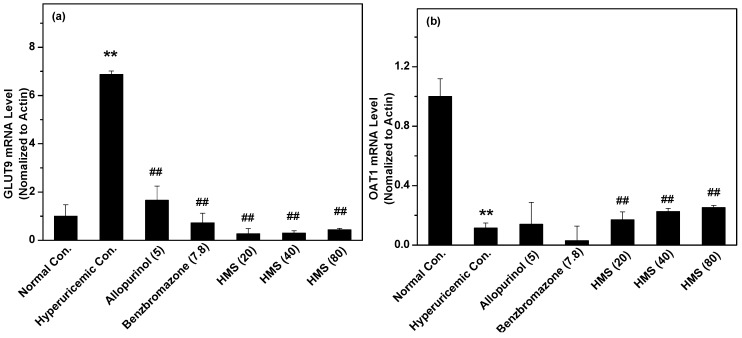
RNA expression levels of GLUT9 (**a**) and OAT1 (**b**). Data were expressed as mean ± SD; n = 3. Statistical analysis by one-way ANOVA followed by two-tailed Student’s *t*-test; ** *p* < 0.01 versus the normal control; ^##^ *p* < 0.01 versus hyperuricemic control.

Since GLUT9 functioned as a key uric acid transporter for reabsorption on the basolateral surface, its RNA expression was recorded. As expected, hyperuricemic control demonstrated elevated GLUT9 RNA expression (Figure 7a), but benzbromarone and HMS at various doses decreased that. OAT1 plays a role in uric acid secretion which lines the basolateral surface of nephron cells and functions for transporting uric acid from blood into nephron cells. Obviously, HMS enhanced its RNA expression (Figure 7b).

**Figure 8:**
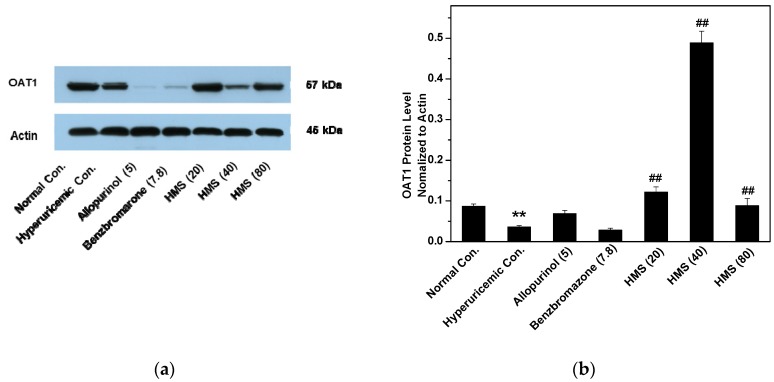
Protein levels OAT1 in kidney: (**a**) Western blot bands; (**b**) the contents determined through densitometry and normalized to actin. Data were expressed as mean ± SD; n = 3. Statistical analysis by one-way ANOVA followed by two-tailed Student’s *t*-test; ** *p* < 0.01 *versus* the normal control; ^##^ *p* < 0.01 *versus* hyperuricemic control.

PO and HX decreased OAT1 proteins (Figure 8). Allopurinol and benzbromarone reduced it further, but HMS at 20 and 80 mg/kg restored OAT1 protein levels.

**Figure 9:**
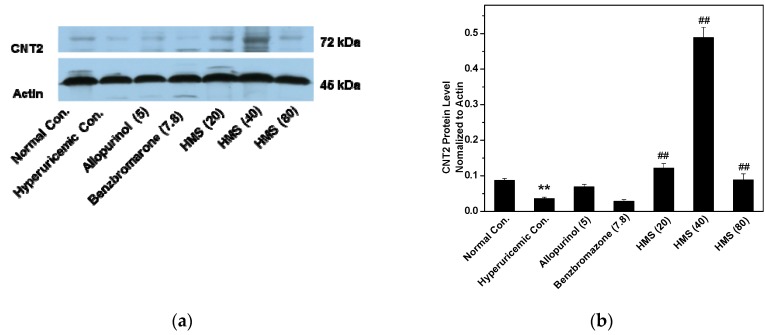
CNT2 protein levels in gastrointestinal tract: (**a**) Western blot bands; (**b**) the contents determined through densitometry and normalized to actin. Data were expressed as mean ± SD; n = 3. Statistical analysis by one-way ANOVA followed by two-tailed Student’s *t*-test; ** *p* < 0.01 versus the normal control; ^##^ *p* < 0.01 versus hyperuricemic control. ** *p* < 0.01 versus the normal control; ^##^ *p* < 0.01 versus hyperuricemic control.

CNT2 is the key transporter impacting SUA since CNT2 transports purines from intakes into blood in gastrointestinal tract. Herein, hyperuricemic control showed lower CNT2 protein level than normal control (Figure 9). Allopurinol restored it, but HMS at three doses enhanced CNT2 protein levels.

## 3. Discussion

Hyperuricemia plays a dominant role in gout development, for which the main treatment is to lowering SUA [24]. The haemostasis of SUA is modulated in three segments in the purine metabolism pathway, consisting of the purine absorption in the gastrointestinal tract, the metabolism into uric acid by XOD and the regulation by the kidney through secretion and reabsorption [25]. In recent years, the prevalence of hyperuricemia has been becoming higher and higher [26], and more youngsters are getting this disease. Thus, searching more effective natural products or compounds of high efficacy and safety is important. In this study, we described the excellent anti-hyperuricemic effect of HMS, which demonstrated comparable efficacy to standard drugs, showing promising perspectives for further development or structural modification. Also its general toxicity was assessed, showing no effects on body weight growth, and some negative effects on liver but not on kidney. Some anti-inflammatory effects were also observed through spleen and thymus changes. Its anti-hyperuricemic mechanisms were explored in vitro and in vivo, and may be ascribed to its inhibition of XOD activities in vitro and in vivo and its up-regulation of OAT1 and down-regulation of GLUT9.

XOD is the key enzyme functioning for converting purines into uric acid in the liver [27], from where then it is distributed into the blood and kidneys, hence, XOD has been a key target focus for several decades for researchers all over the world. Many synthesized compounds or natural products against it were developed or discovered, taking compounds with the core scaffolds of six-membered ring, with bicyclic structure, tricyclic structure and condensed tetracyclic structures as examples [28]. As a typical bicyclic structure, benzophenones with hydroxyls [22] or thiazolidinone groups [23] were synthesized and found to be active against XOD. Specially, the most active one, 2,2′,4,4′-tetrahydroxybenzophenone, exhibited a IC_50_ of 47.59 μM [22] and compound 9m displayed about 76% of the activity of allopurinol [23]. However, it was not clear whether they could reduce SUA in vivo, although they provided a novel skeleton against hyperuricemia. Inspired by this, HMS was docked and then assayed against XOD by us. The docking was performed with a flexible approach. Like a nail, HMS’s two cyclic moieties were buried into the inner tunnel and its two substitutes attached to aromatic moiety sited and occupied the outer tunnel. Thus, its binding to XOD was fastening, even better than oxypurinol, which used as a positive control in docking in many researches. Generally for anti-hyperuricemic research against XOD, allopurinol was used as a positive control in vitro. Herein, the inhibitory activity of HMS was some lower than that of allopurinol against XOD, but also they were within one magnitude.

SUA, serum creatinine and BUN are the key physiochemical parameters for anti-hyperuricemic research, which index its anti-hypericemic efficacy and the important renal function. The abolishment of the hyperuricemic effect of hyperuricemic mice by allopurinol was obviously observed. Importantly, the in vivo anti-hyperuricemic efficacy of HMS was observed of to be near that of allopurinol and benzbromarone. Of note, some uricosuric effect was observed for HMS. To explore the mechanisms of HMS against hyperuricemia in hyperuricemic mice, the livers and serum were collected for in vivo XOD activity assays after drug administration to mice for 7 days. HMS demonstrated a significant XOD inhibitory effect in vivo, which is consistent with the in vitro results. Inhibition against XOD may be a mechanism for the anti-hyperuricemic effect of HMS. Of course, in many patients hyperuricemia is due to gradual kidney function fading, which means that the transportation activities of one or more transporters, such as OAT1 [29] and GLUT9 [30], are abnormal. OAT1 lines on the basolateral side of nephron cells, functioning for elevating uric acid secretion through transporting uric acid into nephron cells from blood, while GLUT9 lines the basolateral surface of nephron cells, functioning for increasing reabsorption of uric acid by transporting uric acid into the blood from nephron cells. The changes in RNA or protein expression of GLUT9 or OAT1 tell us about how HMS influenced the two key uric acid transporters GLUT9 or OAT1. HMS enhanced OAT1 RNA and protein levels. Thus, up-regulation of OAT1 may be a mechanism for the anti-hyperuricemic effect of HMS. HMS down-regulated GLUT9. Thus, down-regulation of GLUT9 may also be a mechanism for the anti-hyperuricmic effect of HMS.

In summary, HMS showed excellent anti-hyperuricemic effects. It demonstrated no negative impact on body weight growth and kidney function. An anti-inflammatory effect was also observed via spleen and thymus changes. The mechanisms of its anti-hyperuricemic effect were explored and it may be ascribed to its significant inhibition activity against XOD, up-regulation of OAT1 and down-regulation of GLUT9.

## 4. Materials and Methods

### 4.1. Materials

Potassium oxalate (PO) and hypoxanthine (HX) for mouse model establishment, allopurinol and benzbromarone as positive control drugs and HMS were obtained from Adamas Reagent Co. (Shanghai, China). Assay kits for uric acid, urea nitrogen and creatinine was supplied by Mindray Medical Corp. (Shenzhen, China). R & D Systems Inc. (Minneapolis, MN, USA) provided XOD Activity Elisa Kits. PCR primers and TRIZOL reagent were offered by Servicebio (Wuhan, China).

### 4.2. Molecular Docking

The molecular docking was performed using a flexible docking software CDOCKER embedded in Discovery Studio (DS) 3.0 program (BIOVIA Co., Ltd., San Diego, CA, USA), where -CDOCKER_ENERGY was used to evaluate the interactions [31]. Salicylate was selected as the center of the active pocket with a radius of 10 Å in the receptor of XOD crystal structure (PDB ID, 1FIQ) [27]. HMS and oxypurinol as positive control were sketched and then energy minimized using the CHARMm force field. Docking was conducted with the default parameters. For obtained poses, -CDOCKER_ENERGY and -CDOCKER_INTERACTION_ENERGY were computed and used to rank them. Pose with the highest -CDOCKER_ENERGY were chosen for further analysis.

### 4.3. XOD Inhibition

XOD inhibition of HMS was assayed sepectrophotometrically at 290 nm by a Multimode Microplate Reader (Thermo Scientific, Waltham, MA, USA) with allopurinol as a positive control as the method reported previously [32]. Briefly, the test compound (50 μL at various concentrations in PBS with pH at 7.5) was co-incubated with XOD (50 μL, 7.5 × 10^−8^ mol L^−1^ in PBS with pH at 7.5) for 15 min in a 96-well microplate; while PBS and allopurinol were exploited as negative and positive controls, respectively. After incubation, the reaction was initiated by adding xanthine (150 μL, 5.0 × 10^−5^ mol L^−1^ in PBS with pH at 7.5). The absorptions at 290 nm were monitored and recorded by the Multimode Microplate Reader. The inhibition (%) = [1 − (slope of reaction kinetics equation obtained by reaction with inhibitor)/(slope of reaction kinetics equation obtained by reaction without inhibitor)] × 100%.

### 4.4. Animals

The experiments with mice were approved by and performed at the Guangdong Institute of Microbiology (GT-IACUC20171016-1). Guangdong Provincial Medical Laboratory Animal Centre (Guangzhou, China) supplied Male SPF Kunming mice (20 ± 2 g), which was divided into seven groups, consisting of normal and hyperuricemic controls, allopurinol and benzbromarone positive controls and drug groups of HMS (n = 10) at three doses. The method reported previously [32] was exploited for establishing the hyperuricemic models by dosing 500 mg/kg HX together with HX 300 mg/kg PO for all groups except normal control.

### 4.5. Drug Administration

Allopurinol and benzbromarone positive controls were administered orally with allopurinol (5 mg/kg) and benzbromarone (7.8 mg/kg), respectively. For HMS drug groups, the mice were orally administered with 20, 40, and 80 mg/kg for each group, respectively.

### 4.6. Uric Acid, XOD, URAT1, OAT1, BUN and Creatinine Assays

Serum and urine were collected for measurements of uric acid, creatinine and urea nitrogen according to the phosphotungstic acid reaction [33], Jaffe reaction [34] and urease reaction [35], respectively. In vivo XOD activity of serum and liver was examined using a commercial ELISA Kit in accordance with manufacture’s protocols after serum and liver were collected following a 7 day experiment. Due to the importance of OAT1 and GLUT9 in uric acid transportation, their RNA expressions were examined via RT-PCR with primers as before [25]. OAT1 and CNT2 proteins were examined by western blotting as before [26] due to the dominant role of CNT2 in purine transportation from gastrointestinal tract into blood. Immunoblotting was assayed using OAT1 (1:2000; OAT1 Antibody, ab135924, Abcam Inc., Cambridge, MA, USA) and CNT2 (1:2000; CNT2 Antibody, BS5670, Bioworld Technology Inc., Louis Park, MN, USA) as well as β-actin (1:2000; β-Actin Antibody, GB12001, Servicebio Co., Wuhan, China) antibodies. The contents were determined through densitometry using Alpha Innotech (AlphaEaseShop, (Alpha Innotech, San Leandro, CA, USA) and normalized to actin of each band. Body weight and inner organ coefficient were collected. 

### 4.7. Statistical Analysis

Data were analysed through one-way analysis of variance (ANOVA) in SPSS (Release 11.5, SPSS Inc., Armonk, NY, USA) and depicted as mean ± standard error. The difference was significant at *p* < 0.05 or 0.01 level (two-tailed Student’s *t*-test).
